# Antimicrobial Activity of Gallium Compounds on ESKAPE Pathogens

**DOI:** 10.3389/fcimb.2018.00316

**Published:** 2018-09-10

**Authors:** Sarah Hijazi, Daniela Visaggio, Mattia Pirolo, Emanuela Frangipani, Lawrence Bernstein, Paolo Visca

**Affiliations:** ^1^Department of Science, Roma Tre University, Rome, Italy; ^2^Terrametrix, Menlo Park, CA, United States

**Keywords:** antibacterial, ESKAPE, gallium maltolate, gallium nitrate, gallium protoporphyrin IX, iron uptake

## Abstract

ESKAPE bacteria are a major cause of multidrug-resistant infections, and new drugs are urgently needed to combat these pathogens. Given the importance of iron in bacterial physiology and pathogenicity, iron uptake and metabolism have become attractive targets for the development of new antibacterial drugs. In this scenario, the FDA-approved iron mimetic metal Gallium [Ga(III)] has been successfully repurposed as an antimicrobial drug. Ga(III) disrupts ferric iron-dependent metabolic pathways, thereby inhibiting microbial growth. This work provides the first comparative assessment of the antibacterial activity of Ga(NO_3_)_3_ (GaN), Ga(III)-maltolate (GaM), and Ga(III)-protoporphyrin IX (GaPPIX), belonging to the first-, second- and third-generation of Ga(III) formulations, respectively, on ESKAPE species, including reference strains and multidrug-resistant (MDR) clinical isolates. In addition to the standard culture medium Mueller Hinton broth (MHB), iron-depleted MHB (DMHB) and RPMI-1640 supplemented with 10% human serum (HS) (RPMI-HS) were also included in Ga(III)-susceptibility tests, because of their different nutrient and iron contents. All ESKAPE species were resistant to all Ga(III) compounds in MHB and DMHB (MIC > 32 μM), except *Staphylococcus aureus* and *Acinetobacter baumannii*, which were susceptible to GaPPIX. Conversely, the antibacterial activity of GaN and GaM was very evident in RPMI-HS, in which the low iron content and the presence of HS better mimic the *in vivo* environment. In RPMI-HS about 50% of the strains were sensitive (MIC < 32) to GaN and GaM, both compounds showing a similar spectrum of activity, although GaM was more effective than GaN. In contrast, GaPPIX lost its antibacterial activity in RPMI-HS likely due to the presence of albumin, which binds GaPPIX and counteracts its inhibitory effect. We also demonstrated that the presence of multiple heme-uptake systems strongly influences GaPPIX susceptibility in *A. baumannii*. Interestingly, GaN and GaM showed only a bacteriostatic effect, whereas GaPPIX exerted a bactericidal activity on susceptible strains. Altogether, our findings raise hope for the future development of Ga(III)-based compounds in the treatment of infections caused by multidrug-resistant ESKAPE pathogens.

## Introduction

ESKAPE species (*Enterococcus faecium, Staphylococcus aureus, Klebsiella pneumoniae, Acinetobacter baumannii, Pseudomonas aeruginosa*, and *Enterobacter* species) are among the most common bacterial pathogens in nosocomial infections, causing extensive morbidity and mortality, especially in critically ill and immunocompromised patients (Rice, 2010). All these species are characterized by a high level of antibiotic resistance (Pendleton et al., 2013), which recently prompted the World Health Organization to list ESKAPE pathogens among the greatest threats to human health, and to boost research on new effective drugs for treatment of antibiotic-resistant infections (World Health Organization, 2017). Among Gram-negative ESKAPE species, *K. pneumoniae, A. baumannii* and *P. aeruginosa* have reached an alarmingly high level of resistance, causing infections which are no longer treatable with conventional antibiotic therapies (Deplano et al., 2005; Elemam et al., 2009; Nowak et al., 2017). Depriving bacteria of essential nutrients, such as iron, is a viable strategy for the development of new antibacterials. Iron is a key nutrient for nearly all forms of life, including bacteria, being a cofactor of many vital enzymes (e.g., those involved in cellular respiration, DNA synthesis, and defense against reactive oxygen species) (Andrews et al., 2003). During infection, bacteria are faced with iron scarcity in body fluids, and must gain access to iron bound to transferrins (e.g., transferrin and lactoferrin) and/or heme-containing proteins (e.g., hemoglobin and myoglobin) (Weinberg, 2009). To counteract iron-limitation, bacteria have developed high-affinity iron-uptake strategies, such as: (i) the production of low-molecular-weight compounds, called siderophores, which bind Fe(III) and actively transport the metal into the cell (Miethke and Marahiel, 2007); (ii) the ability to utilize heme iron, by producing hemophores and/or specific transport systems for heme and heme-binding proteins (Wandersman and Delepelaire, 2004); and (iii) the active transport of Fe(II) through the cytoplasmic membrane *via* the Feo system (Cartron et al., 2006). Given the essential role of iron in bacterial physiology and pathogenicity, iron uptake and metabolism have become attractive targets for the development of new antibacterials (Ballouche et al., 2009; Foley and Simeonov, 2012). In this regard, the ferric iron [Fe(III)] mimetic ion gallium [Ga(III)] has been shown to inhibit the growth of many bacterial and fungal species by interfering with iron-dependent metabolic pathways (Bastos et al., 2010; Minandri et al., 2014). Given the chemical similarity between Fe(III) and Ga(III), microorganisms cannot easily distinguish between these two ions, so that Ga(III) competes with Fe(III) for incorporation into essential proteins and enzymes. However, unlike Fe(III), Ga(III) cannot be reduced under physiological conditions, resulting in the inhibition of several iron-dependent redox pathways (Bernstein, 1998).

**Figure 1:**
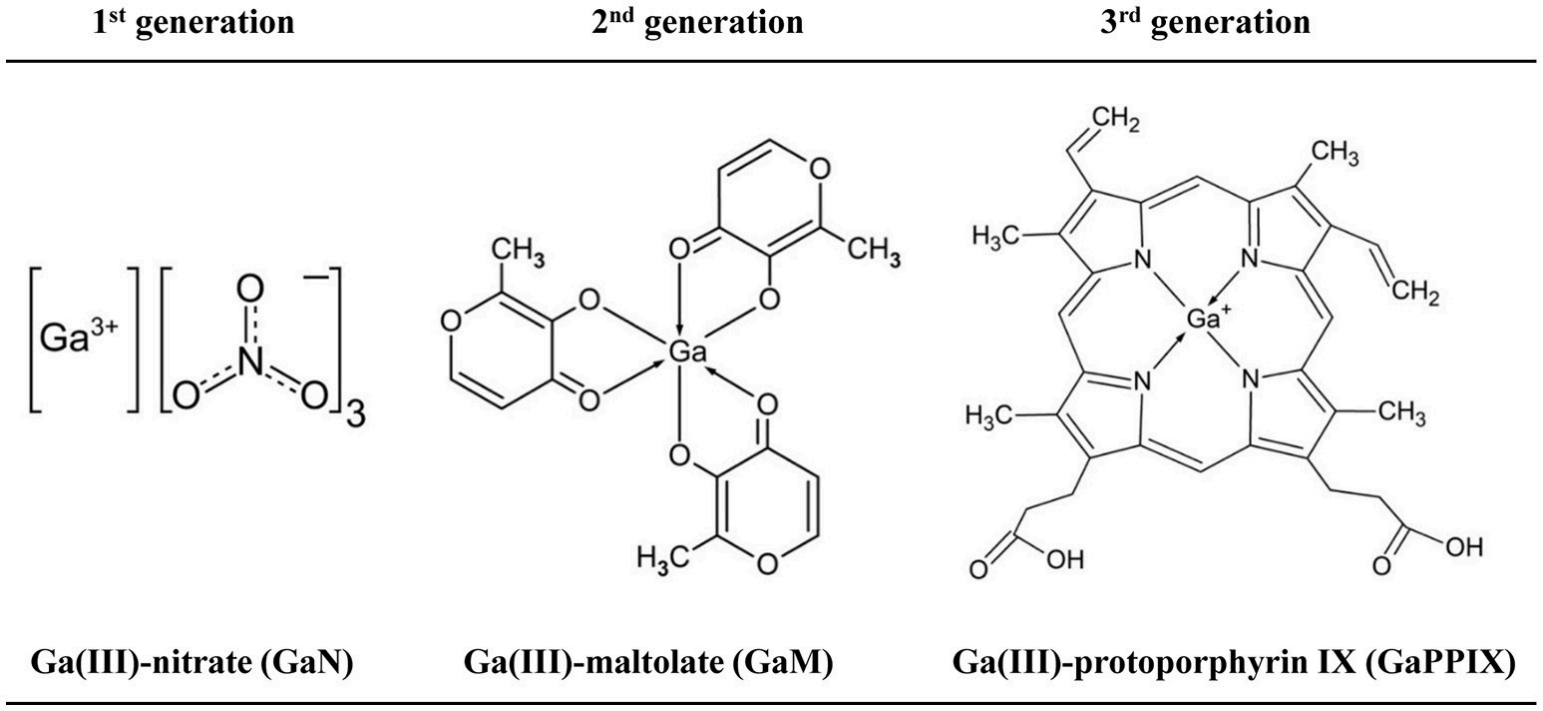
Chemical structures of the three Ga(III) compounds used in this study.

For more than three decades, Ga(III) compounds have been employed as diagnostic tools in medicine. Radioactive ^67^Ga allows localization of malignant cells and inflammatory or infective foci (Edwards and Hayes, 1969). Citrated Ga(NO_3_)_3_ (GaN, brand name Ganite®, Genta, NJ, USA) was approved by the US FDA for the treatment of cancer-associated hypercalcemia, though it is no longer available. Recently, there has been an expansion in the number of Ga(III) compounds showing therapeutic potential, sometimes categorized in first, second, and third generations, and ranging from simple salts such as GaCl_3_ and GaN, through metal-organic complexes such as Ga(III)-maltolate (GaM) (Bernstein et al., 2000) and Ga(III)-protoporphyrin IX (GaPPIX) (Chitambar, 2017) (Figure 1). It is noted that GaN has very low oral bioavailability and must be parenterally administered, whereas GaM has high oral bioavailability and has been safely administered orally to people (Bernstein et al., 2000).

At present, neither standard protocols nor reference media for Ga(III)-susceptibility testing have been defined, though several lines of evidence indicate that iron irreversibly suppresses the antibacterial properties of Ga(III) (Kaneko et al., 2007; Antunes et al., 2012). Moreover, there are no comparative data on the activity of different Ga(III) compounds against ESKAPE species under standard test conditions, representing a major pitfall to the repurposing of Ga(III) as last-resort antibacterial agent.

In this study, the antibacterial activity of three compounds belonging to the first-, second-, and third-generation Ga(III) formulations, i.e., GaN, GaM, and GaPPIX, was tested on ESKAPE pathogens in culture media characterized by different iron content, namely Mueller-Hinton broth (MHB), iron-deprived MHB (DMHB) (Hackel et al., 2018) and RPMI-1640 tissue culture medium supplemented with 10% complement-free human serum (RPMI-HS), to better mimic the *in vivo* environment (Antunes et al., 2012; Thompson et al., 2012; Bonchi et al., 2015). ESKAPE bacteria resulted more susceptible to Ga(III) compounds in RPMI-HS than in MHB and DMHB. However, the presence of serum albumin in RPMI-HS and the type and number of bacterial heme-uptake systems strongly influenced GaPPIX susceptibility. Intriguingly, GaPPIX exerted a bactericidal activity on some strains, whereas GaN and GaM invariably exhibited bacteriostatic effects.

## Materials and methods

### Bacterial strains and culture conditions

Bacterial strains used in this work are listed in Table S1. Bacteria were routinely cultured for 18 h in Tryptic Soy Broth (TSB, Acumedia) with vigorous shaking. When required, tetracycline (Tc), gentamicin (Gm), and 5-bromo-4-chloro-3-indolyl-β-D-galactopyranoside (X-Gal, Sigma) were added to the media. For *A. baumannii*, 50 μg/ml Tc, and 100 μg/ml Gm were used. For *Escherichia coli* 10 μg/ml Gm and 40 μg/ml X-Gal were used. When vitamins (Vit) were required, 19 μg /ml of nicotinic acid (Sigma-Aldrich), and 2 μg /ml of pyridoxal hydrochloride (MERCK) were added to the media. Bovine hemin chloride (Sigma-Aldrich) was freshly prepared in 10 mM NaOH. Bovine serum albumin (BSA, Sigma-Aldrich) was freshly prepared and added to the media at the final concentration of 5 mg/ml.

### Media and Ga(III) compounds

Three media have been used in this study: (i) BBL Mueller Hinton II (Cation-Adjusted) Broth (MHB, Becton Dickinson) was prepared according to the manufacturer's instructions; (ii) DMHB was prepared following the approved CLSI protocol for antimicrobial susceptibility testing (Hackel et al., 2018). Briefly, MHB was treated for 16 h at 4°C with 100 g/l of the metal-chelating Chelex® 100 resin (Bio-Rad) under moderate stirring, then filtered through Whatman no. 1 filter paper and pH adjusted to 7.3. After autoclaving, CaCl_2_ and MgSO_4_ were added to DMHB at the final concentrations of 22.5 and 11.25 μg/ml, respectively; and (iii) RPMI-1640 (Sigma-Aldrich) supplemented with 10% complement-free human serum (RPMI-HS). Human serum was collected from 140 healthy donors, pooled, filtered, and inactivated (30 min, 56°C), as previously described (Antunes et al., 2012). Bulk serum chemistry was: total serum proteins 80 mg/ml; total iron 0.70 μg/ml; ferritin 0.243 μg/ml; transferrin 2.63 mg/ml; total iron binding capacity 4.27 mg/ml (20% transferrin saturation).

Three Ga(III) compounds were used in this study: (i) GaN (Ga(NO_3_)_3_ × 6H_2_O, Sigma-Aldrich; quality tested), freshly prepared as a 100 mM stock solution in water; (ii) GaM (NORAC Pharma, provided by Dr. Bernstein), freshly prepared as a 22 mM stock solution in water; and (iii) GaPPIX (Frontier Scientific), prepared as a 25 mM stock solution in dimethyl sulfoxide (DMSO), and stored at 4°C in the dark.

### Iron content measurement, siderophore production, and β-galactosidase (LacZ) activity assays

The iron concentration of MHB, DMHB, and RPMI-HS was measured by inductively coupled plasma optical emission spectrometry (ICP-OES) using an ICP-OES 710 Varian Spectrometer (Agilent Technologies). Briefly, the medium was mixed with 5% HNO_3_, heated for 1 h at 90°C, and filtered through a Millipore membrane (pore size 0.45 μm) prior to ICP-OES analysis.

Siderophore production was determined by the chrome azurol S-Fe(III)-hexadecyltrimethylammonium bromide method (Schwyn and Neilands, 1987). Activity of the *basA*::*lacZ* reporter gene fusion carried by plasmid pMP220::P*basA* (Antunes et al., 2012) was tested in the reference strain *A. baumannii* ATCC 17978, and β-galactosidase levels were expressed as Miller units (Miller, 1972).

### Susceptibility testing of Ga(III) compounds

The inhibitory activity of Ga(III) compounds on ESKAPE pathogens was assessed by the microdilution method (Clinical Laboratory Standards Institute, 2015), with minor modifications. Bacteria were grown for 18 h in TSB, then washed in saline and diluted to obtain *ca*. 5 × 10^5^ CFU/ml in 200 μl of MHB, DMHB, or RPMI-HS, in the presence of increasing concentrations (0 to 128 μM) of each Ga(III) compound [GaN or GaM or GaPPIX], using 96-well microtiter plates. Plates were incubated for 24 h at 37°C with orbital shaking (110 rpm). The MIC of Ga(III) compounds was determined as the lowest concentration that completely inhibited bacterial growth as detected by the unaided eye (Clinical Laboratory Standards Institute, 2015). To test the effect of Fe(III) and hemin on GaPPIX antibacterial activity, freshly prepared FeCl_3_ (Sigma-Aldrich) or bovine hemin chloride (Sigma-Aldrich) were added at the indicated final concentrations, into 200 μl of MHB inoculated with *ca*. 5 × 10^5^ CFU/ml, in the presence of GaPPIX supplied at the MIC. Microtiter plates were incubated for up to 24 h at 37°C and bacterial growth {optical density at 600 nm [OD_600_]} was periodically measured using SPARK 10M TECAN reader.

The antibacterial activity of GaPPIX on *A*. *baumannii* strains was also assessed by disk diffusion assays. Briefly, 18 h cultures in TSB were washed and diluted in saline to OD_600_ = 0.1, then seeded with a sterile swab on the surface of RPMI-HS supplemented with 15 g/l agar (Acumedia). Sterile 6-mm blank disks (ThermoFisher-Oxoid) soaked with 10 μl of a 15 mM solution of GaPPIX were deposited on the agar surface, and the growth inhibition halo was detected after 18 h incubation at 37°C.

### Time-kill assays

Time-kill kinetic assays of Ga(III) compounds were performed on eleven ESKAPE pathogens according to a previously described procedure (Principe et al., 2009), with minor modifications. Briefly, tubes containing 1 ml of RPMI-HS supplemented with 28 μM of GaN, GaM or GaPPIX were inoculated with bacteria to a density *ca*. 5 × 10^5^ CFU/ml, and incubated at 37°C with gentle shaking (120 rpm). Aliquots were removed at time 0, 3, 6, and 24 h post-inoculation, and serially diluted in saline for determination of viable counts on Luria Bertani (LB) agar plates.

### Identification and cloning of the heme-utilization gene clusters in *A. baumannii*

Previously described oligonucleotides and PCR conditions were used to check the presence of genes belonging to the heme iron-uptake gene cluster 2 (hereafter termed *hemT* cluster), and the heme iron-uptake gene cluster 3 (Antunes et al., 2011), which includes the *hemO* gene, hence named *hemO* cluster (Ou et al., 2015).

The 9,833 bp DNA fragment encompassing eight genes of the *hemO* cluster of ACICU (from ACICU_00873 to ACICU_00880 locus) was obtained by PCR amplification using primers HemO_FW (5′-CATTTGGTTTCCGAGTCTCG-3′) and HemO_RV (5′-CCATGATGCGTACCATGCA-3′). The PCR product was purified by the PCR Clean-Up System (Promega) and blunt-end ligated to the SmaI site of pVRL1 (Lucidi et al., 2018), yielding plasmid pVRL1*hemO*. The pVRL1*hemO* plasmid was introduced in *A. baumannii* ATCC 17978 by electroporation according to published procedures (Yildirim et al., 2016). Transformants were selected on LB agar plates supplemented with 100 μg/ml Gm.

## Results

### DMHB and RPMI-HS are iron-poor media that support the growth of ESKAPE species

For a comparative assessment of the antibacterial effect of the three Ga(III) compounds, a representative collection of ESKAPE species was used, including reference strains and multidrug-resistant (MDR) clinical isolates (Table S1). Since Ga(III) is an Fe(III)-mimetic acting as a metabolic competitor of Fe(III), its antibacterial activity depends on the iron concentration in the test medium, being enhanced by conditions of relative iron scarcity (Minandri et al., 2014). Therefore, both chemical analyses and functional assays were performed to probe iron content and availability in MHB, DMHB, and RPMI-HS media, prior to Ga(III)-susceptibility testing. ICP-OES measurements (Figure S1) showed that the iron concentrations in DMHB (0.43 μM) and RPMI-HS (1.95 μM) were lower than in MHB (3.38 μM). The relatively high iron concentration of RPMI-HS can be ascribed to partially (*ca*. 20%) iron-saturated transferrin in human serum, since only iron traces (0.11 μM) are present in serum-free RPMI-1640 (data not shown). To evaluate whether DMHB and RPMI-HS are perceived by bacteria as iron-poor media, both siderophore production and iron-repressible gene expression were investigated by using *A. baumannii* ATCC 17978 as a biosensor organism. Notably, high siderophore levels were produced in both DMHB and RPMI-HS, as opposed to MHB (Figure S1). Moreover, a transcriptional fusion between the promoter of the iron-repressible *basA* gene and the reporter *lacZ* gene (Antunes et al., 2012) was expressed by *A. baumannii* ATCC 17978 at higher levels in DMHB and RPMI-HS than in MHB (Figure S1). These data indicate that DMHB and RPMI-HS are low-iron media that induce an iron-starvation response during bacterial growth. ESKAPE pathogens share similar iron-mediated regulatory mechanisms of gene expression, all possessing the Ferric uptake regulator protein Fur, which drives the expression of iron-repressible genes, including those for siderophore-biosynthesis (i.e., *basA*). Therefore, it can be assumed that the *basA*::*lacZ* transcriptional fusion provides an indirect estimate of the intracellular iron levels of ESKAPE bacteria grown in different media (Ochsner and Vasil, 1996; Achenbach and Yang, 1997; Haley and Skaar, 2012; Mortensen and Skaar, 2013; Carpenter and Payne, 2014; Latorre et al., 2018). The ability of ESKAPE pathogens to grow in DMHB and RPMI-HS was then tested for the reference strains of each species (Figure S2). For all strains tested, evident growth reduction (12–60%) was observed in DMHB compared with MHB. Addition of 3 μM FeCl_3_ to DMHB (i.e., restoring the iron concentration of MHB before Chelex® 100 treatment) rescued the growth of all strains, except *E*. *faecium* ATCC 19434 and *S. aureus* ATCC 25923 (Figure S2A). For these two species, the residual growth reduction observed in iron-replete DMHB is likely due to the removal of other metabolically relevant metals, besides iron. Moreover, all but one strain grew in RPMI-HS, although at different rates (Figure S2B). The only exception was *E*. *faecium* ATCC 19434, whose growth was rescued by the addition of two vitamins, namely nicotinic acid and pyridoxal hydrochloride. These cofactors were added to RPMI-HS to allow Ga-susceptibility testing of *E. faecium* (Figure S2C). These preliminary experiments allowed us to establish iron-poor culture conditions in conventional media that support the growth of all ESKAPE strains tested, thus being suitable for comparative testing of the antibacterial activity of Ga(III) compounds.

### Susceptibility of ESKAPE pathogens to Ga(III) compounds

**Figure 2:**
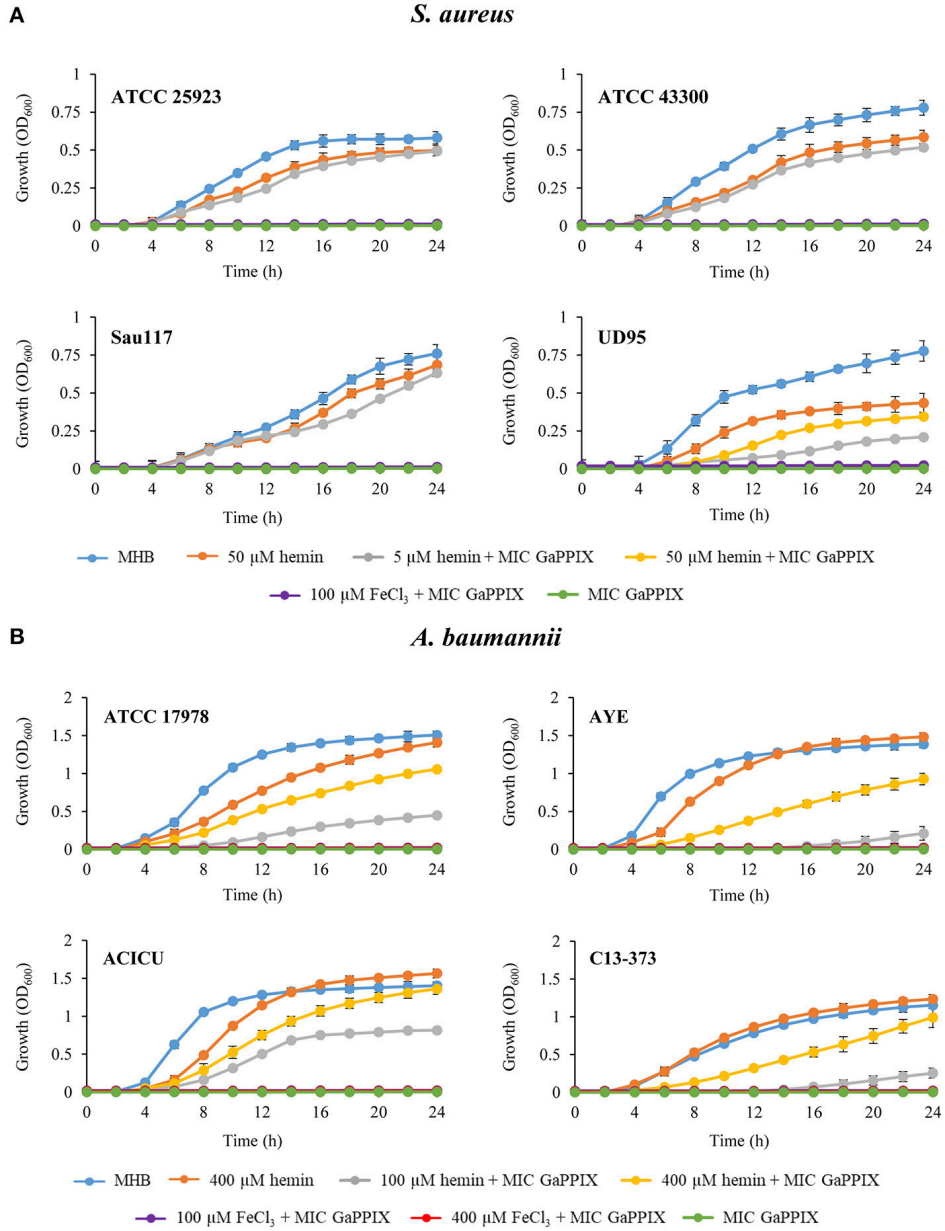
Effect of iron and hemin on the inhibitory activity of GaPPIX. Strains were grown in MHB supplemented or not with GaPPIX at the MIC. Hemin and FeCl_3_ were supplied at different concentrations, either alone or in combination with GaPPIX, as indicated. **(A)** *S. aureus* ATCC 25923, ATCC 43300, Sau117, UD95, and **(B)** *A. baumannii* strains ATCC17978, AYE, ACICU and C13–373. OD_600_ was monitored periodically for up to 24 h. Data are the mean ± standard deviation of triplicate experiments.

The activity of the three Ga(III) compounds was tested on a total of 24 ESKAPE strains in three selected media (Table 1). Arbitrarily assuming the resistance breakpoint at MIC > 32 μM, which roughly corresponds to the peak serum concentration of Ga(III) achievable during human therapy (Bernstein, 1998; Collery et al., 2002), all strains were resistant to the three Ga(III) compounds tested in MHB and DMHB, except *S. aureus* and *A. baumannii*, which were susceptible to GaPPIX (MIC ≤ 32 μM). Notably, the MIC of GaPPIX for *S. aureus* was extremely low in both MHB and DMHB (0.06–0.12 μM). Moreover, no differences in MIC values for *S. aureus* and *A. baumannii* were observed between MHB and DMHB, in spite of their different iron content, and thus of the different iron starvation status of bacteria (Figure S1). This suggests a mechanism of action of GaPPIX that is not responsive to iron. In fact, addition to MHB of 100 μM FeCl_3_, i.e., in excess over GaPPIX, did not abrogate the growth inhibitory effect of GaPPIX for all *S. aureus* and *A. baumannii* strains tested (Figure 2), while 100 μM FeCl_3_ alone, used as control in MHB, did not influence bacterial growth (data not shown). Since GaPPIX is likely to exert its antibacterial effect by acting as a heme analog (Stojiljkovic et al., 1999; Hijazi et al., 2017), we wondered whether hemin might counteract bacterial GaPPIX susceptibility. To verify this hypothesis, several concentrations of hemin (from 5 to 400 μM) were added to MHB together with GaPPIX, provided at the MIC (Table 1). The addition of 5 μM hemin was sufficient to completely abrogate the activity of GaPPIX against all *S. aureus* strains, except *S. aureus* UD95, for which a higher hemin concentration (50 μM) was required (Figure 2A). Conversely, not even the highest hemin concentration tested (400 μM) was able to fully reverse the growth-inhibitory effect of GaPPIX in *A. baumannii* (Figure 2B). Of note, also FeCl_3_ provided at 400 μM (i.e., equimolar with the highest hemin concentration tested) did not rescue the growth of all *A. baumannii* strains tested.

**Table 1:** MIC (μM) of Ga(III) compounds for ESKAPE strains.

**Bacterial strain**	**MHB**			**DMHB**			**RPMI-HS^b^**		
	**GaN**	**GaM**	**GaPPIX**	**GaN**	**GaM**	**GaPPIX**	**GaN**	**GaM**	**GaPPIX**
*E. faecalis* ATCC 29212	>128	>128	>128	>128	>128	>128	>128	64	>128
*E. faecalis* ATCC 700802	>128	>128	>128	>128	>128	>128	>128	128	>128
*E. faecium*^T^ ATCC 19434	>128	>128	>128	>128	>128	>128	>128	64	>128
*E. faecium* BM4147	>128	>128	>128	>128	>128	>128	ND	ND	ND
*S. aureus* ATCC 25923	>128	>128	**0.12**	>128	>128	**0.06**	>128	>128	>128
*S. aureus* ATCC 43300	>128	>128	**0.12**	>128	>128	**0.06**	>128	128	>128
*S. aureus* Sau117^a^	>128	>128	**0.06**	>128	>128	**0.06**	>128	128	>128
*S. aureus* UD95^a^	>128	>128	**0.12**	>128	>128	**0.12**	>128	128	>128
*K. pnemoniae* ATCC 27736	>128	>128	>128	>128	>128	>128	>128	>128	>128
*K. pnemoniae* Kp3^a^	>128	>128	>128	>128	>128	>128	>128	>128	>128
*K. pnemoniae* 17830^a^	>128	>128	>128	>128	>128	>128	**4**	**2**	>128
*K. pnemoniae* 16855^a^	>128	>128	>128	>128	>128	>128	**16**	**16**	>128
*A. baumannii* ATCC 17978	>128	>128	**16**	>128	>128	**16**	**1**	**1**	128
*A. baumannii* AYE^a^	>128	>128	**32**	>128	>128	**32**	**2**	**1**	128
*A. baumannii* ACICU^a^	>128	>128	**16**	>128	>128	**16**	**2**	**1**	**1**
*A. baumannii* C13-373^a^	>128	>128	**32**	>128	>128	**32**	**2**	**1**	**0.25**
*P. aeruginosa* ATCC 15692 (PAO1)	>128	>128	>128	64	>128	>128	**8**	**4**	128
*P. aeruginosa* PA14	>128	>128	>128	64	>128	>128	**16**	**8**	128
*P. aeruginosa* LesB58^a^	>128	>128	>128	>128	>128	>128	**0.5**	**0.5**	**8**
*P. aeruginosa* SP-13^a^	>128	>128	>128	64	>128	>128	**8**	**4**	128
*E. aerogenes*^T^ ATCC 13048	>128	>128	>128	>128	>128	>128	>128	>128	>128
*E. aerogenes* 84-6792	>128	>128	>128	>128	>128	>128	>128	>128	>128
*E.cloacae*^T^ ATCC 13047	>128	>128	>128	>128	>128	>128	>128	>128	>128
*E. cloacae* 78-6303	>128	>128	>128	>128	>128	>128	**8**	**8**	>128

T *, type strain; ND, not determined due to the poor growth*.

a , MDR strain;

b *, only in the case of Enterococcus species, RPMI-HS was supplemented with nicotinic acid and pyridoxine to allow bacterial growth. Arbitrarily assuming the resistance breakpoint at MIC > 32 μM, the MIC values for susceptible isolates are shown in bold type*.

Surprisingly, a dramatic loss of GaPPIX activity was observed in RPMI-HS for *S. aureus* and *A. baumannii* (MICs ≥ 128 μM for 4/4 and 2/4 strains, respectively), as opposed to the activity of GaN and GaM which was enhanced for almost all species tested in RPMI-HS, compared with MHB or DMHB (Table 1). Non-fermenting Gram-negative species, *P. aeruginosa* and *A. baumannii*, showed low MIC values for both GaN and GaM (0.5 μM < MIC < 16 μM) with some intra-species variability. Intriguingly, the hypervirulent *P. aeruginosa* LesB58 (Liverpool strain) showed the lowest MIC values for all Ga(III) compounds, and was the only GaPPIX-sensitive *P. aeruginosa* strain (Table 1). Interestingly, half of *K. pneumoniae* strains and one isolate of *E. cloacae* were also susceptible to GaN and GaM in RPMI-HS (MIC ≤ 16 μM). Moreover, no GaPPIX MIC could be determined in RPMI-HS for all Enterobacteriaceae tested (MIC > 128 μM) (Table 1). Of note, Ga(III) susceptibility was not limited to antibiotic-sensitive ESKAPE strains, but also exerted on MDR clinical isolates (Table 1).

### Bactericidal activity of Ga(III) compounds

**Figure 3:**
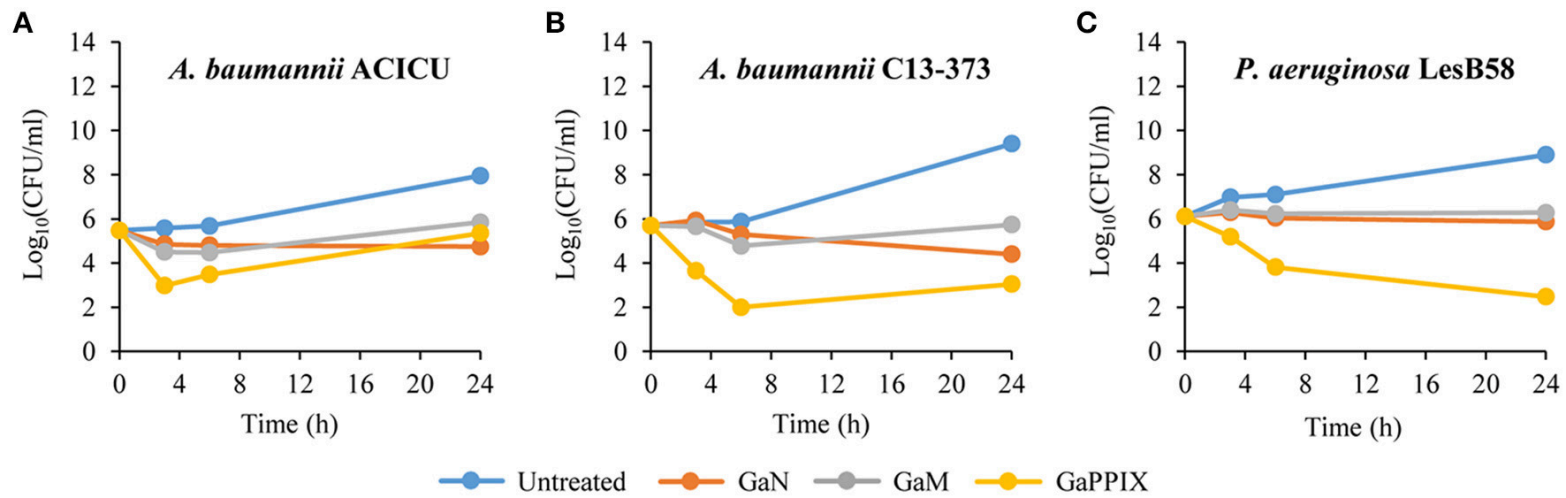
Bactericidal activity of Ga(III) compounds on susceptible strains. GaN, GaM, and GaPPIX time-kill kinetics were determined by plate counts after 0-, 3-, 6-, and 24-h incubation in RPMI-HS supplemented with 28 μM of each Ga(III) compound. **(A)** *A. baumannii* ACICU, **(B)** *A. baumannii* C13-373 and **(C)** *P. aeruginosa* LesB58. Panels show one representative experiment of three independent replicates yielding similar results.

A bactericidal activity has previously been reported for GaN and GaPPIX on *P. aeruginosa* and *A. baumannii*, respectively. However, previous killing assays were conducted in rich laboratory media, which do not mimic the condition encountered *in vivo* by infecting pathogens (Kaneko et al., 2007; Arivett et al., 2015). For this reason, we devised to assess the bactericidal activity of the three Ga(III) compounds in RPMI-HS, i.e., under conditions that trend to mimic biological fluids. Only susceptible strains showing MIC values < 32 μM (Table 1) were selected for bactericidal activity testing of GaN, GaM and GaPPIX, all provided at 28 μM, which corresponds to the peak serum concentration of Ga(III) achievable during human therapy (Bernstein, 1998; Collery et al., 2002). Interestingly, GaN and GaM showed only a bacteriostatic effect (Figure S3, Figure 3), whereas the response to GaPPIX varied among the susceptible strains (Figure 3). The presence of 28 μM GaPPIX caused 2–3 log reduction of viable cells (CFU counts) of *A. baumannii* ACICU, *A. baumannii* C13-373, and *P. aeruginosa* LesB58 after 6 h of incubation at 37°C (Figure 3), indicating a bactericidal effect of GaPPIX in RPMI-HS.

### Serum albumin counteracts the activity of GaPPIX

In many bacterial pathogens, severe iron limitation induces the expression of heme-uptake systems, therefore increasing the susceptibility to GaPPIX (Stojiljkovic et al., 1999; Hijazi et al., 2017). Intriguingly, *S. aureus* was very sensitive to GaPPIX in both MHB and DMHB, while no MIC was determined for GaPPIX in RPMI-HS (Table 1), even though RPMI-HS is iron-poor and induces an iron-starvation response in bacteria (Figure S1). Likewise, *A. baumannii* ATCC 17978 and AYE were much more susceptible to GaPPIX in MHB and DMHB than in RPMI-HS (Table 1). However, while all *S. aureus* strains were resistant to GaPPIX in RPMI-HS (MIC > 128 μM, Table 1), they became extremely sensitive to GaPPIX in RPMI-1640 without HS (MIC ≤ 0.25 μM, Table 2). Likewise, *A. baumannii* ATCC 17978 and AYE became sensitive to GaPPIX in RPMI-1640 without HS (MIC = 8 μM, Table 2), although showing high GaPPIX MIC in RPMI-HS (128 μM, Table 1). These results argue for the presence of HS compound(s) capable of counteracting the antibacterial activity of GaPPIX. Since human serum albumin (HSA), the most abundant plasma protein, binds a variety of ligands including heme (Adams and Berman, 1980), we hypothesized that HSA could bind GaPPIX, due to its chemical similarity with heme, thus impairing its translocation across the cell membrane *via* heme-uptake systems. To test this hypothesis, the susceptibility of *S. aureus* and *A. baumannii* to GaPPIX was determined in MHB and RPMI-1640 supplemented or not with BSA, a protein sharing 76% sequence identity and the same heme-binding properties as HSA (Goncharova et al., 2013). The amount of added BSA was 5 mg/ml, equaling the final concentration of HSA in RPMI-HS. Consistent with our hypothesis, addition of 5 mg/ml BSA to RPMI-1640 dramatically increased (67 to 533 fold) the MICs of GaPPIX on both *S. aureus* and *A. baumannii* (Table 2). A similar effect was also observed upon addition of BSA to MHB (Table 2). Taken together, these results suggest that the poor susceptibility to GaPPIX observed for *S. aureus* and *A. baumannii* in RPMI-HS is due to the presence of HSA which binds GaPPIX and neutralizes its inhibitory effect.

**Table 2:** Effect of BSA on the MIC (μM) of GaPPIX for *S. aureus* and *A. baumannii*.

**Bacterial strains**	**MHB**		**RPMI-1640**	
	**no BSA**	**5 mg/ml BSA**	**no BSA**	**5 mg/ml BSA**
*S. aureus* ATCC 25923	0.12	32	0.12	8
*S. aureus* ATCC 43300	0.12	16	0.12	32
*S. aureus* Sau117	0.06	16	0.25	16
*S. aureus* UD95	0.12	64	0.12	64
*A. baumannii* ATCC 17978	16	> 128	8	128
*A. baumannii* AYE	32	> 128	8	128
*A. baumannii* ACICU	16	> 128	ND	ND
*A. baumannii* C13-373	32	> 128	16	128

*ND, not determined due to the poor bacterial growth*.

### Multiple heme-uptake systems influence *A. baumannii* susceptibility to gappix

**Figure 4:**
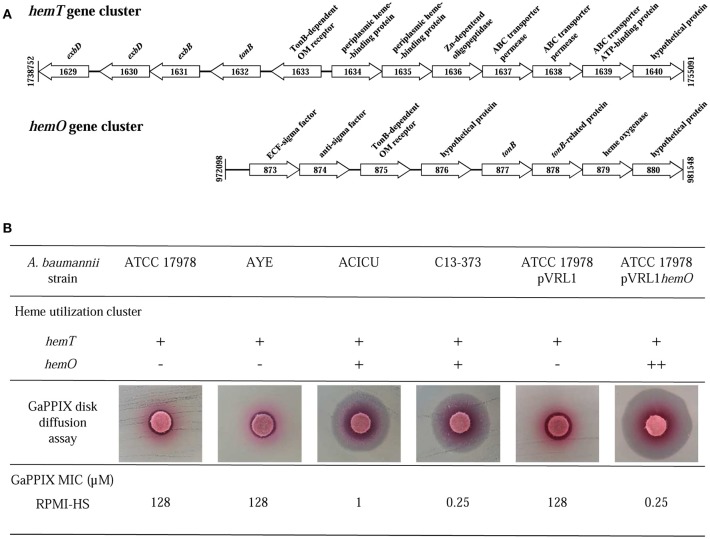
Influence of heme-uptake systems on *A. baumannii* susceptibility to GaPPIX. **(A)** Schematic representation of the two gene clusters for heme uptake, *hemT* and *hemO*, of *A. baumannii* ACICU. Coding sequences are shown as arrows (not to scale) oriented according to the predicted direction of transcription. **(B)** Top; presence (+) or absence (-) of heme clusters in different *A. baumannii* strains (++, overexpression from the multicopy plasmid pVRL1*hemO*). Middle; GaPPIX disk diffusion assay in RPMI-HS agar plates inoculated with different *A. baumannii* strains. Bottom; MICs of GaPPIX for different *A. baumannii* strains in RPMI-HS.

The wide range of GaPPIX susceptibility observed among *A. baumannii* isolates led us to hypothesize that the presence of different GaPPIX-uptake capabilities could be the source of this variability. GaPPIX is known to exploit heme-uptake routes to enter bacterial cells (Stojiljkovic et al., 1999; Hijazi et al., 2017), and all *A. baumannii* strains sequenced so far, including ATCC 17978, AYE and ACICU, possess the *hemT* heme-uptake cluster (homolog of the iron-uptake gene cluster 2 in Antunes et al., 2011). Interestingly, *A. baumannii* ACICU possesses an additional heme-uptake cluster, named *hemO* (iron-uptake gene cluster 3 in Antunes et al., 2011) (Figure 4A). Since *A. baumannii* ACICU showed an extremely low GaPPIX MIC (1 μM), we hypothesized that the presence of *hemO* could be responsible for the increased GaPPIX susceptibility, likely providing a more efficient GaPPIX uptake route. Indeed, PCR analysis revealed the presence of both *hemO* and *hemT* clusters in *A. baumannii* C13-373 (data not shown), another strain showing very low GaPPIX MIC (0.25 μM), similar to *A. baumannii* ACICU (Figure 4B). To shed more light on the relationship between heme uptake and the antibacterial activity of GaPPIX in *A. baumannii*, the whole *hemO* cluster of strain ACICU (9,833 bp), was cloned and expressed *in trans* in *A. baumannii* ATCC 17978. GaPPIX susceptibility of *A. baumannii* ATCC 17978 expressing the whole *hemO* cluster from plasmid pVRL1*hemO* or harboring the empty vector (pVRL1) was then assessed in both solid and liquid RPMI-HS (Figure 4B). In RPMI-HS agar plates, a much larger growth inhibition halo was observed around the GaPPIX-soaked disk for *A. baumannii* ATCC 17978 (pVRL1*hemO*), compared with *A. baumannii* ATCC 17978 (pVRL1), indicating that expression of the *hemO* cluster from a multicopy plasmid *in trans* greatly increases *A. baumannii* ATCC 17978 susceptibility to GaPPIX (Figure 4B). As expected, the presence of the empty vector pVRL1 did not affect the susceptibility of *A. baumannii* ATCC 17978 to GaPPIX (Figure 4B). In line with the results of the disk diffusion assay, MIC data confirmed that *A. baumannii* ATCC 17978 (pVRL1*hemO*) is more susceptible to GaPPIX than *A. baumannii* ATCC 17978 (pVRL1), the former showing a MIC of 0.25 μM in RPMI-HS (Figure 4B). Altogether, these data indicate that the susceptibility of *A. baumannii* to GaPPIX is increased by the presence of the *hemO* gene cluster.

## Discussion

Antimicrobial resistance has become one of the most challenging problems of the healthcare system. The spread of antimicrobial-resistant pathogens has dramatic repercussions on mortality and morbidity rates, hence on global medical costs (Friedman et al., 2016). ESKAPE pathogens rank among the most prevalent causative agents of healthcare-associated infections, and pose a serious therapeutic challenge due to their resistance to available antibiotics (Boucher et al., 2009). However, while new antibiotics are urgently needed, the pipeline of antibiotic discovery is almost dry (Luepke and Mohr, 2017). Iron uptake and metabolism are regarded as druggable targets for new antibacterial strategies (Ballouche et al., 2009; Foley and Simeonov, 2012). In this context, the iron mimetic metal Ga(III) has been shown to inhibit bacterial growth, by interfering with iron-dependent metabolic pathways (Minandri et al., 2014).

In this work, we report the first comparative evaluation of the antibacterial properties of three Ga(III) compounds on ESKAPE species in conventional susceptibility testing media characterized by different iron concentrations and nutrient compositions.

Our data demonstrate that the bacterial susceptibility to Ga(III) compounds varies among species and among strains within the same species, and is influenced by the iron concentration and nutrient composition of the medium. Ga(III)-susceptibility tests conducted in MHB and DMHB showed elevated MIC values (> 32 μM) for all species, except *S. aureus* and *A. baumannii*, which were sensitive to GaPPIX only. Conversely, Ga(III) testing in RPMI-HS gave a better response, with an overall higher susceptibility to GaN and GaM for *K. pneumoniae, A. baumannii, P. aeruginosa*, and *E. cloacae* (MIC ≤ 16 μM for 50 to 100% of strains tested). Among the ESKAPE bacteria tested, aerophilic species, which preferentially adopt a respiratory metabolism, namely *A. baumannii* and *P. aeruginosa*, were in general more susceptible to Ga(III) in RPMI-HS than fermenting species, such as Enterobacteriaceae, *S. aureus* and enterococci. This is probably due to Ga(III)-dependent impairment of iron-demanding processes, such as respiration and response to oxidative stress (Stojiljkovic et al., 1999; Hijazi et al., 2017). Moreover, GaN and GaM showed a similar spectrum of activity, although GaM was more potent than GaN in 9 out of 13 isolates for which a MIC could be determined. Previous experiments in a mouse burn wound model infection are in line with this observation, given that much lower GaM concentrations were needed to prevent *P. aeruginosa* and *A. baumannii* proliferation, compared with GaN (DeLeon et al., 2009). The solubility of GaM in both water and lipids, allowing for the penetration of cell walls and membranes, as opposed to the lack of lipophylicity of GaN, probably accounts for much of the difference in biologic effects between the two compounds (Bernstein et al., 2000; DeLeon et al., 2009). Although the presence of non-selective entrance routes for Ga(III) cannot be excluded, Ga(III) mainly exploits iron-uptake systems to enter bacterial cells (Minandri et al., 2014). Therefore, the variable susceptibility to GaN and GaM observed for *K. pneumoniae* and *E. cloacae* in RPMI-HS could be explained by the variable number and type of iron-acquisition systems in these two species (Podschun et al., 1992; Koczura and Kaznowski, 2003) and perhaps their varying growth rates. This holds true also in *P. aeruginosa*, where siderophores, either pyoverdine (PVD) or pyochelin (PCH), have opposite effects on Ga(III) activity; while PCH shuttles Ga(III) into *P. aeruginosa* cells, PVD sequesters it away in the periplasmic space, therefore protecting bacterial cells from Ga(III)-mediated toxicity (Kaneko et al., 2007; Frangipani et al., 2014).

GaPPIX deserves a special comment. As documented for various bacterial species, GaPPIX exerts its activity when transported into the cell, implying that the presence and/or expression level of heme-uptake genes have a major impact on GaPPIX activity (Stojiljkovic et al., 1999; Hijazi et al., 2017). The *A. baumannii hemO* gene cluster encodes a very efficient heme-utilization system, responsible for an increased translocation of GaPPIX in the cell (de Léséleuc et al., 2014). Results presented here suggest a major role for the *hemO* system also in GaPPIX susceptibility, given that only strains possessing both *hemT* and *hemO* gene clusters were severely inhibited by GaPPIX in RPMI-HS (MIC 0.25–1.0 μM), whereas those possessing only the *hemT* cluster were not (MIC > 128 μM) (Tables 1, 2). Notably, this phenomenon was only observed in RPMI-HS, where GaPPIX is bound by HSA, but not in MHB or DMHB where no albumin is present. This suggests that gene products of the *A. baumannii hemO* cluster efficiently withdraw HSA-bound GaPPIX and deliver it to its intracellular targets. On the other hand, the HemT and Isd heme-uptake systems, which are present in *A. baumannii* and *S. aureus*, respectively (Ascenzi et al., 2015), appear by themselves unable to confer GaPPIX susceptibility in RPMI-HS (but not in MHB and DMHB), probably because these two systems cannot efficiently extract HSA-bound GaPPIX for transport into the cell. These observations are in line with a previous report showing that the addition of 10% HS to MHB caused a 3-fold increase of the GaPPIX MIC for *A. baumannii* (Arivett et al., 2015). Intriguingly, the activity of GaPPIX against *S. aureus* and *A. baumannii* was independent of the iron content of the medium, given that: (i) it was similar in MHB and DMHB media, irrespective of their different iron content, and, (ii) amendment of MHB with an exceedingly high FeCl_3_ concentration did not neutralize the antibacterial activity of GaPPIX (Figure 2), in agreement with previous reports (Stojiljkovic et al., 1999; Arivett et al., 2015). Interestingly, the addition of hemin partially reversed the antibacterial activity of GaPPIX in MHB, albeit in one *S. aureus* and in all *A. baumannii* strains tested not even a molar excess of hemin completely abrogated the effect of GaPPIX (Figure 2). This means that incorporation of GaPPIX in vital bacterial enzymes is only in part reversed by competition with hemin. This observation could have significant clinical implications, since the release of heme and/or iron from red blood cells and iron-binding proteins during inflammatory processes is unlikely to undermine the antibacterial activity of GaPPIX *in vivo*. These observations, together with the previously reported low toxicity of GaPPIX (Stojiljkovic et al., 1999; Arivett et al., 2015; Chang et al., 2016), support the potential use of GaPPIX as a therapeutic option to treat some bacterial infections. It should be noted that, different from GaN and GaM which exploit multiple routes to enter bacteria (Minandri et al., 2014), making the selection of Ga(III)-resistant cells less likely to occur compared with conventional antibiotic treatments (Ross-Gillespie et al., 2014), GaPPIX enters the cell through specialized heme-uptake systems (Stojiljkovic et al., 1999; Hijazi et al., 2017). While this could imply more frequent emergence of GaPPIX-resistant cells through loss of heme (hence GaPPIX) uptake capabilities, the preferential use of heme as iron source by bacterial pathogens *in vivo* argues against a dispensable role of these systems during infection.

In conclusion, we have determined suitable test conditions to assess the antibacterial activity of Ga(III) compounds *in vitro*. The presence of human serum (HS) in RPMI-HS reduces iron availability thanks to the presence of transferrin, thereby providing a more realistic milieu for testing the antibacterial activity of iron-mimetic compounds. Moreover, in RPMI-HS the presence of serum albumin, which interferes with GaPPIX but not of GaN or GaM, indicates that, among the three Ga(III)-compounds tested, the FDA-approved GaN and the orally active GaM were the most effective under conditions that mimic the *in vivo* environment, i.e., in RPMI-HS. With respect to the clinical repositioning of Ga(III) as an antibacterial agent, one should consider that the recommended dosing regimen of citrated GaN for the treatment of cancer patients (200 to 300 mg/m^2^ body surface area, i.v. administration) ensures a peak serum concentration of Ga(III) of *ca*. 28 μM (Bernstein, 1998; Collery et al., 2002). Notably, we found that in RPMI-HS much lower GaN concentrations are needed to inhibit the growth of *A. baumannii, P. aeruginosa* and some Enterobacteriaceae. Ongoing clinical trials on patients with cystic fibrosis (IGNITE study, ClinicalTrials.gov Identifier: NCT02354859) will provide important insights into *P. aeruginosa* inhibition during i.v. GaN treatment of chronic lung infection, hence on the actual potential of Ga(III) as an antibacterial agent. It is also noted that topical or other localized means of administration can readily provide millimolar levels of Ga(III) to sites of infection, including burn-associated infections (DeLeon et al., 2009). In fact, the topic use of Ga(III)-citrate has been shown to improve healing, reduce inflammation and favor reepithelization in a murine wound model of *K. pneumoniae* infection (Thompson et al., 2015).

Interestingly, we found that pre-existing resistance to multiple antibiotics in MDR strains did not compromise Ga(III) susceptibility, likely as a consequence of Ga(III) molecular targets (iron-binding proteins) being different from those of common antibiotics, toward which resistance has been selected. In conclusion, Ga(III) could represent a drug of last resort to combat infections sustained by otherwise untreatable pan-resistant bacteria.

## Author contributions

DV and PV conceived and designed the experiments. SH and MP performed the experiments. SH, DV, EF, and PV analyzed the data. SH wrote the draft manuscript. SH, DV, MP, EF, LB, and PV revised the manuscript.

### Conflict of interest statement

LB holds several patents for possible applications of GaM in human and veterinary medicine and is affiliated with a company (Gallixa LLC) that would like to obtain regulatory approval for topical gallium maltolate as a therapeutic agent. LB did not participate in data collection for this study. The remaining authors declare that the research was conducted in the absence of any commercial or financial relationships that could be construed as a potential conflict of interest.
