# Supplementary material for: Antimicrobial Activity of Gallium Compounds on ESKAPE Pathogens
Source: Front Cell Infect Microbiol. 2018 Sep 10;8:316. doi: 10.3389/fcimb.2018.00316 (PMC6139391; doi:10.3389/fcimb.2018.00316)
Supplement: Supplementary file 1 [file Table_1.DOCX]

***Supplementary Material***

**Antimicrobial activity of gallium compounds on ESKAPE pathogens**

**Sarah Hijazi^1^, Daniela Visaggio^1^, Mattia Pirolo^1^, Emanuela Frangipani^1^, Lawrence Bernstein^2^ and Paolo Visca^1*^**

^1^*Department of Science, Roma Tre University, Rome, Italy*

^2^*Terrametrix, Menlo Park, CA 94025, USA*

***Correspondence**: Prof. Paolo Visca

Department of Science,

Roma Tre University

Viale G. Marconi 446

00146 Rome, Italy

[paolo.visca@uniroma3.it](mailto:paolo.visca@uniroma3.it)

**Table S1.** List of bacterial strains used in this study.

| Bacterial species | Strain | Country | Year | Source | Resistance | Reference |
| --- | --- | --- | --- | --- | --- | --- |
| *Enterococcus faecalis* | ATCC 29212 | ns | ns | urine | ns | American Type Culture Collection |
| *E. faecalis* | ATCC 700802 | USA | 1987 | blood | VAN (*vanB*), GEN | Sahm et al., 1989 |
| *Enterococcus faecium*^T^ | ATCC 19434 | ns | ns | unknown | ns | Schleifer and Kilpper-Balz, 1984 |
| *E. faecium* | BM4147 | ns | ns | human clinical | VAN (*vanA*) | Bugg et al., 1991 |
| *Staphylococcus aureus* | ATCC 25923 | USA | 1945 | human clinical | susceptible | American Type Culture Collection |
| *S. aureus* | ATCC 43300 | USA | ns | human clinical | MRSA | American Type Culture Collection |
| *S. aureus* | Sau117 | Italy | 2010 | skin infection | MDR | Monaco et al., 2013 |
| *S. aureus* | UD95 | Italy | 2011 | human clinical | MDR | This work |
| *Klebsiella pnemoniae* | ATCC 27736 | ns | ns | human clinical | ns | American Type Culture Collection |
| *K. pnemoniae* | Kp3 | Italy | 2011 | blood | MDR | Villa et al., 2014 |
| *K. pnemoniae* | 17830 | Italy | 2008 | sacral ulcer | MDR | Garcia-Fernandez et al., 2010 |
| *K. pnemoniae* | 16855 | Italy | 2008 | wound | MDR | Garcia-Fernandez et al., 2010 |
| *Acinetobacter baumannii* | ATCC 17978 | France | 1951 | blood | SXT | Smith et al., 2007 |
| *A. baumannii* | AYE | France | 2001 | urine | MDR | Poirel et al., 2003 |
| *A. baumannii* | ACICU | Italy | 2005 | spinal fluid | MDR | Iacono et al., 2008 |
| *A. baumannii* | C13-373 | Italy | 2007 | blood | MDR | Migliavacca et al., 2013 |
| *Pseudomonas aeruginosa* | ATCC 15692 (PAO1) | ns | ns | infected wound | ns | American Type Culture Collection |
| *P. aeruginosa* | PA14 | USA | 1995 | burn | susceptible | Rahme et al., 1995 |
| *P. aeruginosa* | LesB58 | UK | 1996 | sputum | MDR | Cheng et al., 1996 |
| *P. aeruginosa* | SP-13 | Italy | ns | blood | MDR | Bonchi et al., 2015 |
| *Enterobacter aerogenes*^T^ | ATCC 13048 | USA | ns | sputum | ns | Bascomb et al., 1971 |
| *E. aerogenes* | 84-6792 | Italy | 2016 | blood | susceptible | This work |
| *Enterobacter cloacae*^T^ | ATCC 13047 | USA | ns | spinal fluid | susceptible | Hormaeche and Edwards, 1960 |
| *E. cloacae* | 78-6303 | Italy | 2016 | blood | susceptible | This work |

Abbreviations: GEN, gentamicin; MDR, multidrug resistant; MRSA, methicillin-resistant *Staphylococcus aureus*; SXT, trimethoprim-sulfamethoxazole; VAN, vancomycin; ns, not specified.

^T^ type strain.

**References:**

Bascomb, S., Lanpage, S. P., Willcox, W. R., and Curtis, M. A. (1971). Numerical Classification of the Tribe *Klebsielleae*. *J. Gen. Microbiol*. 66, 279-295. doi: [10.1099/00221287-66-3-279](https://doi.org/10.1099/00221287-66-3-279)

Bonchi, C., Frangipani, E., Imperi, F. and Visca, P. (2015). Pyoverdine and proteases affect the response of *Pseudomonas aeruginosa* to gallium in human serum. *Antimicrob. Agents Chemother*. 59, 5641-5646. doi: 10.1128/AAC.01097-15

Bugg, T. D. H., Wright, G. D., Dutka-Malen, S., Arthur, M., Courvalin, P., and Walsh, C. T. (1991). Molecular basis for vancomycin resistance in *Enterococcus faecium* BM4147: biosynthesis of a depsipeptide peptidoglycan precursor by vancomycin resistance proteins VanH and VanA. *Biochem.* 30, 10408-10415. doi: 0.1021/bi00107a007

Cheng, K., Smyth, R. L., Govan, J. R., Doherty, C., Winstanley, C., Denning, N., et al. (1996). Spread of beta-lactam resistant *Pseudomonas aeruginosa* in a cystic fibrosis clinic. *Lancet* 348, 639-642 doi: [10.1016/S0140-6736(96)05169-0](https://doi.org/10.1016/S0140-6736(96)05169-0)

García-Fernández, A., Miriagou, V., Papagiannitsis, C. C., Giordano, A., Venditti, M., Mancini, C., et al. (2010). An ertapenem-resistant extended-spectrum-β-lactamase-producing *Klebsiella* *pneumoniae* clone carries a novel OmpK36 porin variant. *Antimicrob. Agents Chemother.* 54, 4178-4184. doi: 10.1128/AAC.01301-09

Hormaeche, E. and Edwards, P. R. (1960). Proposal for the rejection of the generic name *Cloaca* Castellani and Chalmers, and proposal of *Enterobacter* as a generic name with designation of the type species and its type culture*. Int. J. Syst. Evol. Microbiol*. 10, 75-76. doi: [10.1099/0096266X-10-2-75](https://dx.doi.org/10.1099/0096266X-10-2-75)

Iacono, M., Villa, L., Fortini, D., Bordoni, R., Imperi, F., Bonnal, R. J., et al. (2008). Whole-genome pyrosequencing of an epidemic multidrug-resistant *Acinetobacter baumannii* strain belonging to the european clone II group. *Antimicrob. Agents Chemother.* 52, 2616-2625. doi: 10.1128/AAC.01643-07

Migliavacca, R., Espinal, P., Principe, L., Drago, M., Fugazza, G., Roca, I., et al. (2013). Characterization of resistance mechanisms and genetic relatedness of carbapenem-resistant *Acinetobacter baumannii* isolated from blood, Italy. *Diagn. Microbiol. Infect. Dis.* 75, 180-186. doi: 10.1016/j.diagmicrobio.2012.11.002

Monaco, M., Pedroni, P., Sanchini, A., Bonomini, A., Indelicato, A., and Pantosti, A. (2013). Livestock-associated methicillin-resistant *Staphylococcus aureus* responsible for human colonization and infection in an area of Italy with high density of pig farming. *BMC Infect. Dis.* 13:258. doi: 10.1186/1471-2334-13-258

Poirel, L., Menuteau, O., Agoli, N., Cattoen, C., and Nordmann, P. (2003). Outbreak of extended-spectrum β-lactamase VEB-1-producing isolates of *Acinetobacter baumannii* in a french hospital. *J. Clin. Microbiol.* 41, 3542-3547. doi: [10.1128/JCM.41.8.3542-3547.2003](https://dx.doi.org/10.1128%2FJCM.41.8.3542-3547.2003)

Rahme, L. G., Stevens, E. J., Wolfort, S. F., Shao, J., Tompkins, R. G., and Ausubel, F. M. (1995). Common virulence factors for bacterial pathogenicity in plants and animals. *Science* 30, 1899-1902. doi: 10.1126/science.7604262

Sahm, D. F., Kissinger, J., Gilmore, M. S., Murray, P. R., Mulder, R., Solliday, J. et al. (1989). *In vitro* susceptibility studies of vancomycin-resistant *Enterococcus faecalis*. *Antimicrob. Agents Chemother.* 33,1588-1591.

Schleifer, K. H. and Kilpper-Balz, R. (1984). Transfer of *Streptococcus faecalis* and *Streptococcus faecium* to the genus *Enterococcus* norn. rev. as *Enterococcus faecalis* comb. nov. and *Enterococcus faecium* comb. *Nov. Int. J. Syst. Evol. Microbiol.* 34, 31-34.

Smith, M. G., Gianoulis, T. A., Pukatzki, S., Mekalanos, J. J., Ornston, L. N., Gerstein, M., et al. (2007). New insights into *Acinetobacter baumannii* pathogenesis revealed by high-density pyrosequencing and transposon mutagenesis. *Genes Dev*. 21, 601-614. doi: [10.1101/gad.1510307](https://doi.org/10.1101/gad.1510307)

Villa, L., Feudi, C., Fortini, D., García-Fernández, A., and Carattoli, A. (2014). Genomics of KPC-producing *Klebsiella pneumoniae* sequence type 512 clone highlights the role of RamR and ribosomal S10 protein mutations in conferring tigecycline resistance. *Antimicrob. Agents Chemother*. 58, 1707-12. doi: [10.1128/AAC.01803-13](https://doi.org/10.1128/AAC.01803-13)
