# Supplementary material for: Antimicrobial Activity of Gallium Compounds on ESKAPE Pathogens
Source: Front Cell Infect Microbiol. 2018 Sep 10;8:316. doi: 10.3389/fcimb.2018.00316 (PMC6139391; doi:10.3389/fcimb.2018.00316)
Supplement: Supplementary file 2 [file Image_1.PDF]

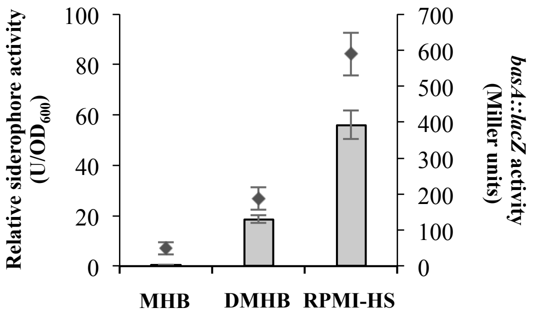

**Total iron concentration (µM)**	3.38	0.43	1.95

Figure S1. Probing the iron content of Ga(III)-susceptibility test media. Siderophore production (grey bars), and activity of the *basA*::*lacZ* iron-regulated reporter gene fusion (black diamonds) in *A. baumannii* ATCC 17978, grown in MHB, DMHB, or RPMI-HS for 24 h. Data are the mean ± standard deviation of triplicate experiments. The iron content of media, as determined by ICP-OES, is also reported.
