# Supplementary material for: Antimicrobial Activity of Gallium Compounds on ESKAPE Pathogens
Source: Front Cell Infect Microbiol. 2018 Sep 10;8:316. doi: 10.3389/fcimb.2018.00316 (PMC6139391; doi:10.3389/fcimb.2018.00316)
Supplement: Supplementary file 3 [file Image_2.PDF]

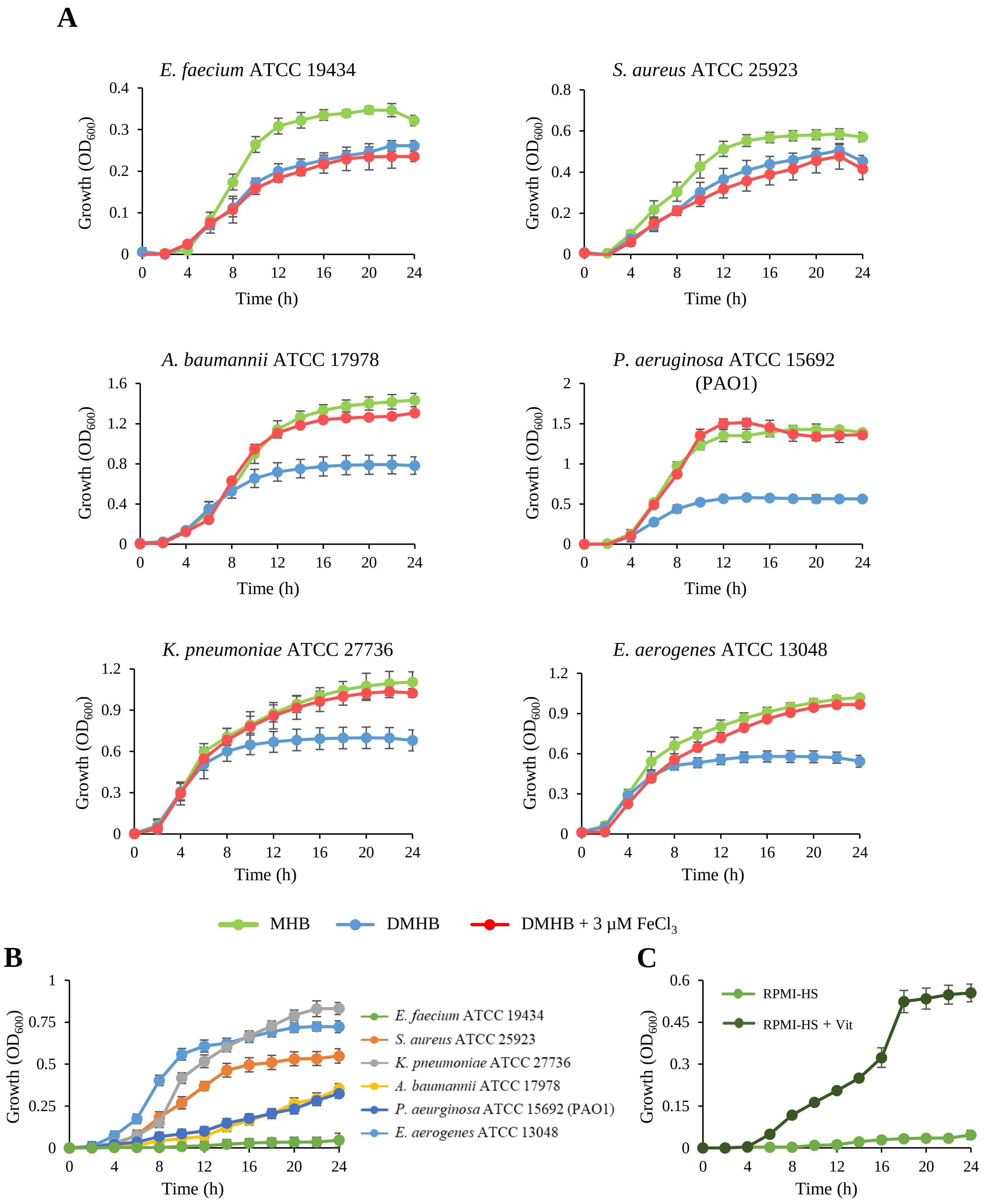

Figure S2. Growth profiles of ESKAPE reference strains in MHB, DMHB, and RPMI-HS. **(A)** Bacterial strains were grown for 18 h at 37 °C in TSB, diluted in saline and inoculated (5 x 10^5 CFU/ml) into 96-well microtiter plates containing MHB, or DMHB supplemented or not with 3.3 µM $FeCl_3$. **(B)** Same as **(A)**, but using RPMI-1640 supplemented with 10 % HS (RPMI-HS). **(C)** *E. faecium* growth rescue in RPMI-HS supplemented with 19 µg/ml of nicotinic acid and 2 µg/ml of pyridoxal hydrochloride (Vit). OD_{600} was monitored periodically for up to 24 h. Data are the means ± standard deviation of triplicate experiments.
