# Supplementary material for: Antimicrobial Activity of Gallium Compounds on ESKAPE Pathogens
Source: Front Cell Infect Microbiol. 2018 Sep 10;8:316. doi: 10.3389/fcimb.2018.00316 (PMC6139391; doi:10.3389/fcimb.2018.00316)
Supplement: Supplementary file 4 [file Image_3.PDF]

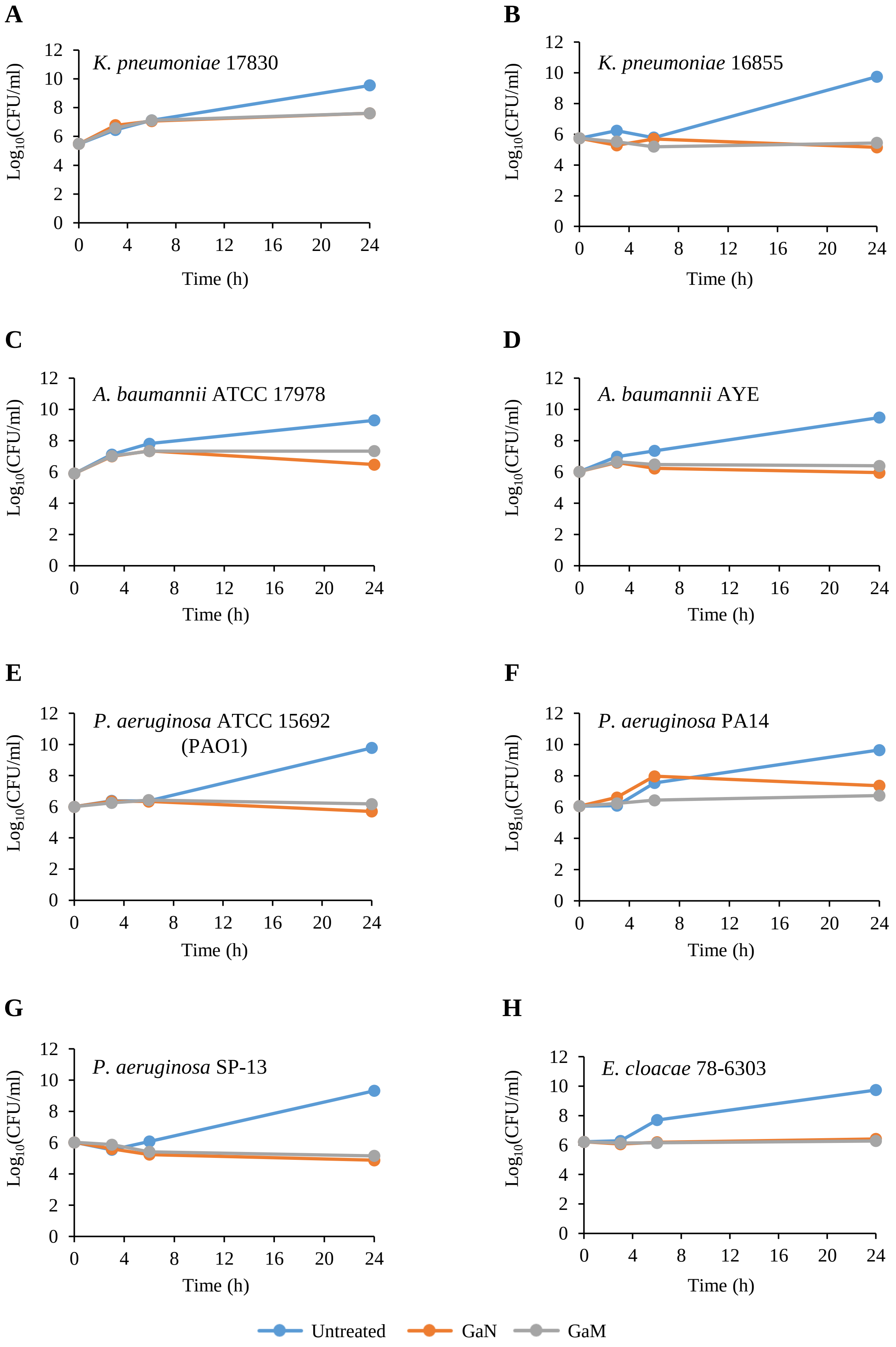

Figure S3. GaN and GaM time-kill assays. GaN and GaM time-kill kinetics were determined after 0-, 3-, 6- and 24-h incubation in RPMI-HS supplemented with 28 µM of GaN or GaM. **(A)** *K. pneumoniae* 17830, **(B)** *K. pneumoniae* 16855, **(C)** *A. baumannii* ATCC 17978, **(D)** *A. baumannii* AYE, **(E)** and *P. aeruginosa* PAO1, **(F)** *P. aeruginosa* PA14, **(G)** *P. aeruginosa* SP-13, and **(H)** *E. cloacae* 78-6303. Panels show one representative experiment of three independent replicates yielding similar results.
